# Clinical Dilemma of Positive Histologic Chorioamnionitis in Term Newborn

**DOI:** 10.3389/fped.2014.00027

**Published:** 2014-04-04

**Authors:** Alain Cuna, Laleh Hakima, Yun-An Tseng, Bianca Fornier, Shahidul Islam, Maria Lyn Quintos-Alagheband, Poonam Khullar, Barry Weinberger, Nazeeh Hanna

**Affiliations:** ^1^Department of Pediatrics, Winthrop University Hospital, Mineola, NY, USA; ^2^Department of Pathology, Winthrop University Hospital, Mineola, NY, USA; ^3^Department of Biostatistics, Winthrop University Hospital, Mineola, NY, USA; ^4^Department of Pediatrics, Rutgers-Robert Wood Johnson Medical School, New Brunswick, NJ, USA

**Keywords:** placental examination, infection, newborn

## Abstract

**Background:** Although histologic chorioamnionitis (HCA) is known to be associated with poor outcomes in preterm infants, its clinical significance among term infants is not clearly known.

**Objectives:** To investigate the utility of HCA in determining early onset clinical sepsis (EOCS) among term newborns.

**Methods:** The incidence of HCA and EOCS in term infants born during 2008–2009 was evaluated in a single center retrospective study (*n* = 3417). The predictive value of HCA for determining EOCS in term infants admitted to the neonatal intensive care unit (NICU) for suspected sepsis (*n* = 388) was quantified. Outcome of otherwise healthy term infants in the nursery with HCA was also investigated.

**Results:** Overall, 11% of term infants with HCA also had EOCS. HCA was associated with increased risk for EOCS (OR 2.6, 95% confidence interval 1.6–4.2, *P* < 0.001) among term infants admitted to the NICU for suspected sepsis. No cases of EOCS were found among otherwise well-appearing infants in the nursery with HCA. Multiple logistic regression analysis indicated that addition of HCA does not increase the power of a model combining C-reactive protein (CRP) and immature to total neutrophil ratio in determining EOCS.

**Conclusion:** Although HCA in term infants is associated with EOCS, it did not improve the ability of CRP and immature to total neutrophil ratio to predict EOCS. Routine placental examination may not contribute to the diagnosis of EOCS in term infants.

## Introduction

Despite advances in obstetrical management including intrapartum antibiotic therapy, early onset clinical sepsis (EOCS) continues to be an important cause of neonatal morbidity and mortality (1). The potential for serious adverse outcomes has led to low thresholds for the evaluation of suspected EOCS in newborns, possibly resulting in unnecessary testing and treatment. The combination of perinatal risk factors, infant signs and symptoms, and laboratory markers are often used to identify infants with suspected EOCS (2). Placental examination is another resource that can provide important insights in the diagnosis and management of sick newborns (3, 4). Histologic chorioamnionitis (HCA), defined by the presence of polymorphonuclear leukocytes within the fetal membranes (5), is a common finding in preterm deliveries (6–8) that has been linked to poor outcomes including bronchopulmonary dysplasia (9), intraventricular hemorrhage (10), periventricular leukomalacia, cerebral palsy (11, 12), and EOCS (13, 14). The majority of these morbidities are thought to be related to the fetal inflammatory response and production of pro-inflammatory cytokines associated with exposure to chorioamnionitis *in utero* (15–17).

Histologic chorioamnionitis is also identified on placental examination after up to 23.6% of full-term deliveries (5), but its clinical significance and predictive value for EOCS in term infants are not known. Clinicians are often faced with the conundrum of an apparently healthy full-term baby with an unexpected placental examination report suggestive of HCA. Another dilemma is whether placental pathology reports should be used to guide the diagnosis or management of infants admitted to the neonatal intensive care unit (NICU) for suspected sepsis. In this study, we examined whether HCA is associated with EOCS in term infants, and whether it improves the prediction of EOCS in these patients.

## Subjects and Methods

This single center, retrospective cohort study was conducted at Winthrop University Hospital, a tertiary center in Mineola, NY, USA with approximately 5000 deliveries annually. Institutional review board approval was obtained prior to the start of this study.

We identified from the neonatal and pathology databases term infants born between January 1, 2008 and December 31, 2009 who had placental histologic examination available (*n* = 3417). Data regarding presence or absence of HCA were obtained from placental pathology reports, and HCA positive cases were reviewed by blinded pathologists for staging of HCA based on the Redline classification system (18). Placental histologic examination is not routinely done for term babies in our hospital unless they are admitted to the NICU or ordered at the discretion of the attending obstetrician based on recommendations by the College of American Pathologists (19).

To investigate the predictive value of HCA in infants evaluated for EOCS, we identified term infants born during the study period that were admitted to the NICU for suspected sepsis. Indications for admission were either maternal risk factors (including chorioamnionitis, group B streptococcus (GBS) colonization with inadequate intrapartum antibiotic prophylaxis, prolonged rupture of membranes) or signs and symptoms suggestive of EOCS (including respiratory distress, oxygen requirement, unexplained hypoglycemia, temperature instability, poor feeding, perinatal depression). Data were obtained regarding the clinical course of infants, including complete blood counts (CBC), immature to total neutrophils (IT) ratio, serial C-reactive protein (CRP) levels, blood culture, and duration of antibiotic therapy. CBC and blood culture were obtained immediately upon admission to the NICU for suspected EOCS. Repeat CBCs were done at 24 and 48 h after admission. Serial CRPs were obtained at 12, 24, and 48 h of life. Abnormal CRP was defined as at least two consecutive CRP values ≥10 g/L and a high IT ratio was defined as ≥0.20.

Our primary outcome of interest is diagnosis of EOCS, which is defined as a composite of proven sepsis (positive blood culture) or probable sepsis (clinical signs and laboratory findings suggestive of infection without positive blood culture) (20). Infants were excluded from analysis if (1) diagnosis of sepsis and subsequent antibiotic treatment of ≥7 days was based solely on the presence of known HCA (*n* = 11) or (2) data obtained from chart review were incomplete (*n* = 13).

### Statistical analysis

Descriptive statistics were presented as proportions. Fisher’s exact test was used to evaluate association between sepsis and HCA. Stepwise multiple logistic regression models were used and areas under receiver operator characteristic (ROC) curves were computed using the method of Hanley and McNeil (21, 22) and DeLong et al. (23) to identify the best model for sepsis. Positive predictive value (PPV) and negative predictive value (NPV) of HCA were calculated with their respective 95% confidence interval (CI) using exact binomial proportion (24). All calculations were performed utilizing SAS 9.3 (SAS Institute, Cary, NC, USA); results were considered statistically significant when *P* < 0.05.

## Results

### Study population

**Figure 1:**
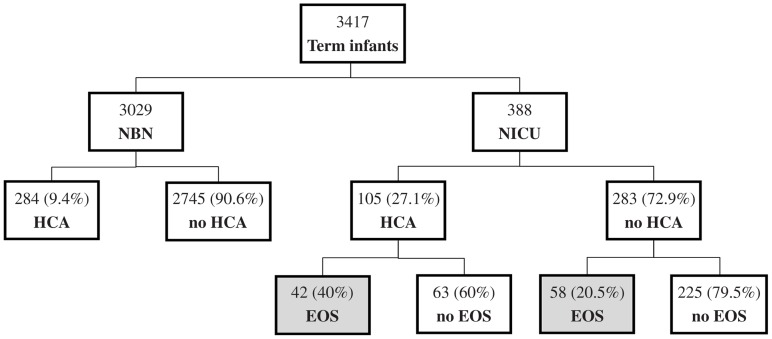
**Of the 3029 asymptomatic term infants admitted to the normal newborn nursery (NBN), 284 (9.4%) had evidence of HCA**. None of those asymptomatic infants developed EOCS. Of the 388 infants admitted to the NICU for suspected sepsis, HCA was noted in 105 infants (27.1%). The incidence of EOCS in infants admitted to the NICU with suspected EOCS was higher in HCA group vs. no HCA group (40 vs. 20.5%, *P* < 0.001).

Of the 3417 term infants with available histopathologic examination during the study period, 3029 were asymptomatic with no risk factors for sepsis and were admitted to the nursery. The remaining 388 infants had risk factors and/or clinical signs suspicious for sepsis and were admitted to the NICU. Among infants admitted to the nursery, 284 (9.4%) had HCA and none had EOCS. Of the infants admitted to the NICU for suspected sepsis, 105 (27.1%) had HCA and 100 (25.8%) had EOCS. The incidence of EOCS was observed to be higher among infants with HCA compared to infants without HCA (40 vs. 20.5%, *P* < 0.001, Fisher’s exact test) (Figure 1).

### Infants with HCA were more likely to have abnormal CRP and IT ratios

Of the 388 infants admitted to the NICU for suspected EOCS, more infants with HCA had abnormal CRP (42.3 vs. 24%, *P* < 0.001) and IT ratio (20.2 vs. 6.4%, *P* < 0.001) compared to infants without HCA. Blood culture was positive in only three patients (one with HCA and two without HCA) (Table 1).

**Table 1:** **Sepsis work-up characteristics of infants with vs. without HCA**.

	HCA		Unadjusted OR (95% CI)	*P*-value
	Yes, *n* = 105 (27%)	No, *n* = 283 (73%)	
High CRP^a^	44 (42.3)	68 (24)	2.3 (1.4–3.7)	<0.001
High IT ratio^b^	21 (20.2)	18 (6.4)	3.7 (1.9–7.3)	<0.001
Blood culture positive	1 (0.96)	2 (0.71)		NS

*Data shown as *n* (%) or odds ratio (95% confidence interval). *P*-values refer to Fisher’s exact test. CRP, C-reactive protein; HCA, histologic chorioamnionitis; IT, immature to total neutrophil; NS, not significant*.

*^a^High CRP was defined as any value ≥10 g/L*.

*^b^High IT ratio was defined as any value ≥0.20*.

### HCA is associated with EOCS but provides little benefit in determining EOCS in term infants

Using Fisher’s exact test, HCA was found to be associated with EOCS (OR 2.6, 95% CI = 1.6–4.2, *P* < 0.001). The PPV of HCA for EOCS was 40% (95% CI = 31–50%) and the NPV was 80% (95% CI = 74–84%).

Stepwise multiple logistic regression analysis was then used to evaluate models of predicting EOCS, utilizing areas under ROC curves to compare predictive power. A model using CRP alone yielded an area under the curve (AUC) of 0.774. A model using both CRP and IT ratio combined significantly improved predictive power with AUC of 0.796 (*P* < 0.0001). A model adding HCA to CRP and IT ratio did not significantly improved predicting power (Table 2).

**Table 2:** **Best model for predicting EOCS using stepwise multiple logistic regression analysis**.

Model	AUC
CRP alone	0.774
CRP + IT ratio	0.796 (Final model)
CRP + IT ratio + HCA	0.808^a^

*AUC, area under the curve; CRP, C-reactive protein; EOCS, early onset clinical sepsis; HCA, histologic chorioamnionitis; IT, immature to total neutrophil*.

*^a^Not a significant increase in AUC*.

Subgroup analysis was also done to evaluate whether classifying HCA based on severity using the Redline classification would improve its ability to predict EOCS. Of the 105 with HCA, 6 were classified as stage 1, 96 as stage 2, and 3 as stage 3. There was no association between increasing staging of HCA and incidence of EOCS (*P* = 0.507).

## Discussion

In this study, we sought to investigate the clinical utility of HCA among term infants in determining EOCS. We observed in our population that HCA was more common among term infants with suspected EOCS compared to well-infants in the nursery. We also observed that infants with HCA were more likely to have abnormal CRP and IT ratios, and subsequently diagnosed with EOCS. Although there was an association between HCA and EOCS among term infants, we showed that addition of HCA to CRP and IT ratio provided little benefit in determining EOCS in term infants.

Several studies have consistently associated placental finding of HCA with early onset sepsis. In a retrospective study of more than 300 preterm infants, presence of HCA on placental examination was associated with an almost sevenfold increased risk for sepsis (OR 6.9, 95% CI = 2.2–20, *P* = 0.001) (14). Another retrospective study with almost 1300 preterm infants demonstrated a similar increased risk for sepsis when HCA was present (OR 5.5, 95% CI = 2.2–13.7) (15). Likewise, a recent prospective study involving 301 preterm infants showed a more than twofold increased risk for sepsis in HCA positive preterm infants (OR 2.22, 95% CI = 1.02–4.83, *P* = 0.04) (25). Most of these studies however were done in preterm infants.

In our study, 389 of the 3417 term placentas (11.4%) examined during the study period had HCA, suggesting that HCA is also a common finding among term infants. This is consistent with other studies that have reported the incidence of HCA in term placentas anywhere from 11.5% (26) to as high as 57.3% (27). The wide range of reported incidence in the literature is likely the result of differing criteria used in determining placentas to be sent for histopathologic examination. Despite guidelines from the College of American Pathologists regarding indications for submission of placentas for pathological examination (19), there remains a wide range of difference in the recommended vs. observed practice of submitting placentas for examination (28–31).

The majority of HCA cases (284/389, 73%) were found among well-appearing term infants, representing almost 10% of infants admitted to the nursery. With current recommendations for discharge as early as 48 h following an uncomplicated vaginal delivery, a number of infants with HCA are likely already discharged home from the nursery by the time placental pathology reports are available. Currently, there is unclear evidence on how to approach this clinical dilemma. Our study is reassuring, in that, none of the well-appearing term infants in the nursery with HCA had EOCS. This suggests that in otherwise healthy-appearing infants, an isolated finding of HCA does not warrant further diagnostic testing or treatment with antibiotics. It also further highlights the importance of relying on good clinical assessment in identifying newborn infants at risk for early onset sepsis.

The remaining HCA cases (105/389, 27%) were found among term infants with signs and/or risk factors suspicious for sepsis. Our study demonstrated that although HCA was associated with EOCS in this population, it did not provide any additional benefit in predicting risk for EOCS beyond that derived from the current recommended diagnostic testing (consisting of blood culture, CBC, and serial CRP). Though results of placental examination are not available at the time when the decision to start antibiotics is made, they are often reported at around the time when clinicians determine whether to continue antibiotic therapy for presumed EOCS. Our study suggests that it is safe and reasonable to not include placental finding of HCA in decision-making regarding continued treatment for suspected EOCS. It is important to emphasize that our study does not suggest that placental examinations are not needed in term infants. Indeed, a number of important findings can be gleaned from the placenta that could prove useful for other diagnoses, including perinatal asphyxia, intrauterine growth restriction, and twin to twin transfusion.

Our study is consistent with that of Roberts et al., who correlated histologic and microbiological evaluation of term placentas. He observed that although 34% (67/195) of placentas had evidence of HCA, only 4% (8/195) had evidence of any infection based on culture and PCR analysis, demonstrating that HCA in term infants is often a result of non-infectious inflammatory process (32). In another study, Torricelli et al. looked at placentas from 395 term pregnancies and found no correlation between finding of HCA and adverse neonatal outcome, including sepsis (26).

There are a number of limitations to our study. The lack of follow-up, especially of well-appearing term infants in the nursery with isolated HCA, prevented us from determining if any infant subsequently developed sepsis after discharge. The inclusion of only term infants with available placental examination, although necessary to determine HCA, likely resulted in a selection bias toward a higher risk population, as term placentas are generally not submitted for examination unless indicated (for cases of maternal disease, pregnancy complications, fetal/neonatal conditions, etc.). The rate of HCA among well-infants in our study may thus be an overestimation and likely does not reflect the general term population. The inclusion of both proven and probable sepsis in our definition of EOCS is also an important limitation. Although the most objective evidence for diagnosis of sepsis is microbiological isolation of bacteria from culture, false-positives from contamination by skin flora and false-negatives from small sampling volumes or pre-treatment with antibiotics are not uncommon. There are thus no simple criteria that can be used for the diagnosis of sepsis in newborns, and the combination of clinical and laboratory findings suggestive of sepsis despite negative cultures have been considered an acceptable surrogate (1). It is also important to note that data on maternal GBS status and intrapartum chemoprophylaxis, although important risk factors for early onset sepsis, were not included in the multivariate analysis involving HCA and EOCS. Because of strict guidelines and algorithms that adhere to the recommendations by Centers of Disease Control and Prevention and American Academy of Pediatrics, almost all term deliveries in our institution had GBS screening performed; and almost all eligible mothers had received appropriate intrapartum chemoprophylaxis. It is thus unlikely that inclusion of maternal GBS status and intrapartum chemoprophylaxis data in the multivariate analysis would have changed the results of our study.

In conclusion, our study demonstrates that although HCA is associated with EOCS, its presence in placental examination adds little to the current practice of clinical observation and limited laboratory evaluation in detecting and managing early onset sepsis in term infants. Routine placental examination may not contribute to the management of early onset sepsis in term infants.

## Conflict of Interest Statement

The authors declare that the research was conducted in the absence of any commercial or financial relationships that could be construed as a potential conflict of interest.
